# Period 2 Regulates CYP2B10 Expression and Activity in Mouse Liver

**DOI:** 10.3389/fphar.2021.764124

**Published:** 2021-11-23

**Authors:** MengLin Chen, Min Chen, Danyi Lu, Yi Wang, Li Zhang, Zhigang Wang, Baojian Wu

**Affiliations:** ^1^ College of Pharmacy, Jinan University, Guangzhou, China; ^2^ Institute of Molecular Rhythm and Metabolism, Guangzhou University of Chinese Medicine, Guangzhou, China; ^3^ Department of Intensive Care Unit, First Affiliated Hospital of Jinan University, Guangzhou, China

**Keywords:** PER2, Cyp2b10, REV-ERBα, cyclophosphamide, drug metabolism

## Abstract

CYP2B10 is responsible for metabolism and detoxification of many clinical drugs. Here, we aimed to investigate a potential role of Period 2 (PER2) in regulating expression of hepatic CYP2B10. Regulatory effects of PER2 on hepatic expression of CYP2B10 and other enzymes were determined using *Per2-*deficient mice with exons 4-6 deleted (named *Per2* ^*Del4-6*^ mice). *In vitro* and *in vivo* metabolic activities of CYP2B10 were probed using cyclophosphamide (CPA) as a specific substrate. Regulatory mechanism was investigated using luciferase reporter assays. Genotyping and Western blotting demonstrated loss of wild-type *Per2* transcript and markedly reduced PER2 protein in *Per2* ^*Del4-6*^ mice. Hepatic expression of a plenty of drug-metabolizing genes (including *Cyp2a4/2a5*, *Cyp2b10, Ugt1a1, Ugt1a9, Ugt2b36, Sult1a1* and *Sult1e1*) were altered (and majority were down-regulated) in *Per2* ^*Del4-6*^ mice*.* Of note, *Cyp2b10, Ugt1a9* and *Sult1a1* were three genes considerably affected with reduced expression. Decreased expression of CYP2B10 was translated to reduced metabolism and altered pharmacokinetics of CPA as well as attenuated CPA hepatotoxicity in *Per2* ^*Del4-6*^ mice. Positive regulation of CYP2B10 by PER2 was further confirmed in both Hepa-1c1c7 and AML-12 cells. Based on luciferase reporter assays, it was shown that PER2 regulated *Cyp2b10* transcription in a REV-ERBα-dependent manner. REV-ERBα was negatively regulated by PER2 (increased REV-ERBα expression in *Per2* ^*Del4-6*^ mice) and itself was also a repressor of CYP2B10. In conclusion, PER2 positively regulates CYP2B10 expression and activity in mouse liver through inhibiting its repressor REV-ERBα.

## Introduction

Drug metabolism (biotransformation) is a main component of pharmacokinetics, profoundly affecting drug efficacy and toxicity (Yan et al., 2018). Drug metabolism reactions, mediated by drug-metabolizing enzymes (DMEs), have been historically divided into two types, namely, phase I and phase II reactions. In phase I reactions (also known as functionalization reactions), DMEs such as cytochromes P450 (CYPs) introduce polar chemical moieties to drug molecules. The modified molecules are then converted to more hydrophilic and excretable metabolites in phase II reactions catalyzed by conjugating DMEs such as UDP-glucuronosyltransferases (UGTs) and sulfotransferases (SULTs). Human CYP superfamily contains 18 families consisting of 57 members (Elfaki et al., 2018). Human UGTs include 22 isoenzymes and are classified into four families (i.e., UGT1, UGT2, UGT3, and UGT8) (Mano et al., 2018). Enzymes from UGT1A and 2B sunfamilies (with a total of 16 members) are the main contributors to drug glucuronidation (Rowland et al., 2013). To date, there are four families of SULT enzymes in humans, namely SULT1, SULT2, SULT4, and SULT6. Enzymes of SULT1 and SULT2 (with a total of 12 members) play a leading role in drug sulfonation (Allali-Hassani et al., 2007).

DMEs such as CYP enzymes are distributed in many tissue and organs, particularly in the drug-metabolizing organs liver, kidney, and small intestine (Renaud et al., 2011). Of CYPs, the members from CYP1 to CYP4 families are of most importance as they metabolize >55% of FDA-approved drugs (Saravanakumar et al., 2019). Mouse CYP2B10 (CYP2B6 in humans) is a member of CYP2B subfamily and primarily expressed in the liver, where it accounts for about 10% of the total microsomal CYP pool (Ekins et al., 1998; Ekins et al., 1999). CYP2B10 is responsible for the metabolism and detoxification of many clinical drugs including cyclophosphamide (CPA) and bupropion (Turpeinen and Zanger, 2012). Expression of CYP2B10 is known to be regulated by a number of nuclear receptors such as pregnane X receptor (PXR), constitutive androstane receptor (CAR), glucocorticoid receptors (GR), and vitamin D receptor (VDR) (Beigneux et al., 2002; Pascussi et al., 2004).

Period 2 (PER2) is a central component of the mammalian circadian clock machinery. It acts as a negative regulator of circadian clock through a direct interaction with the transcriptional activator CLOCK/BMAL1 heterodimer (Langmesser et al., 2008). Phosphorylation by casein kinase 1δ/ε (CK1δ/ε) is a key step that determines the stability of the PER2 protein and therefore the period of the circadian rhythms in mammals (Narasimamurthy et al., 2018). A mutation in CK1-phosphorylating site of PER2 (S662G) has been identified as a determiner of human familial advanced sleep phase syndrome (Vanselow et al., 2006; Xu et al., 2007). PER2 is also involved in the regulation of many physiological and pathological processes such as neurobiological activities (Kim et al., 2018), metabolic homeostasis (Zani et al., 2013), thermogenesis (Chappuis et al., 2013), and tumourigenesis (Mteyrek et al., 2016). Moreover, it has been found that PER2 regulates the hepatotoxicity of drugs such as acetaminophen in mice probably via modulating the expression of CYP1A2 and CYP2E1 (Kakan et al., 2011; Ge et al., 2021). However, whether and how PER2 regulates CYP2B10 remain unknown.

Interestingly, *Per2* ^*−/−*^ mice show decreases in serum triglycerides and free fatty acids coupled with increases in hepatic triglycerides and free fatty acids, indicating that PER2 is involved in lipid metabolism (Kettner et al., 2016). In addition, Heintza’s work illustrates that the repression or inhibition of CYP2B (e.g., CYP2B10) may exacerbate metabolic disorders and cause obesity by perturbing fatty acid metabolism, suggesting a role of CYP2B in lipid homeostasis (Heintz et al., 2019). These findings indicate a potential link between PER2 and CYP2B, particularly CYP2B10.

In the present study, we aimed to investigate a potential role of PER2 in regulating expression of hepatic CYP2B10. Regulatory effects of PER2 on hepatic expression of CYP2B10 and other DMEs were determined using *Per2-*deficient mice with exons 4-6 deleted. mRNAs and proteins were quantified by qPCR and Western blotting, respectively. *In vitro* and *in vivo* metabolic activities of CYP2B10 were probed using CPA as a specific substrate. Regulation of CYP2B10 by PER2 was validated in Hepa-1c1c7 and AML-12 cells. Regulatory mechanism was investigated using luciferase reporter assays. We for the first time demonstrated that PER2 positively regulates CYP2B10 expression and activity in mouse liver through inhibiting its repressor REV-ERBα.

## Materials and Methods

### Materials

Cyclophosphamide (CPA) was purchased from J&K Scientific (Beijing, China). O-methylhydroxylamine (OMHA) was obtained from Steraloids (Wilton, NH). Nicotinamide adenine dinucleotide phosphate (NADPH), uridine diphosphoglucuronic acid (UDPGA), alamethicin, and 3′-phosphoadenosine-5′-phosphosulfate (PAPS) were purchased from Sigma-Aldrich (St. Louis, MO). Propofol and propofol glucuronide were purchased from TargetMol (Shanghai, China). Galangin was obtained from Weikeqi Biotech (Sichuan, China). Assay kits for ALT (alanine aminotransferase), AST (aspartate aminotransferase) and GSH (glutathione) were purchased from Jiancheng Bioengineering Institute (Nanjing, China). Anti-PER2 (ab180655) and anti-GAPDH antibodies (ab9485) were purchased from Abcam (Cambridge, MA). Anti-CYP2B10 (TA504328) and anti-SULT1A1 (TA501951) antibodies were obtained from OriGene (Rockville, MD). Anti-UGT1A9 (bs-4224R) antibody was purchased from Bioss (Beijing, China). Anti-REV-ERBα antibody (AB10130) was purchased from Sigma-Aldrich (St Louis, MO). All primary antibodies were diluted with 5% BSA at a ratio of 1:1,000. The secondary antibody was purchased from Huaan Biotechnology (Hangzhou, China) and diluted with 5% skim milk at a ratio of 1:5,000. siPer2 (siRNA targeting *Per2*) and siNC (a negative control for siPer2) were obtained from TranSheep Biotech (Shanghai, China). pRL-TK vector was purchased from Promega (Madison, WI).

### Mice

*Per2* heterozygotes (on a C57BL/6 background) were obtained from Cyagen Biosciences (Guangzhou, China) (Supplementary Material S1). Homozygotes with exons 4-6 deleted (named *Per2* ^*Del4-6*^ mice) were generated by inter-crossing heterozygous mice. *Per2*-deficient mice and their wild-type littermates were housed and maintained under a 12 h light/12 h dark cycle (lights on at 6:00 AM (= Zeitgeber time 0/ZT0) and lights off at 6:00 PM (= ZT12)), with free access to food and water. Animal experimental procedures were approved by Institutional Animal Care and Use Committee of Guangzhou University of Chinese Medicine (Appr. date: 2020-11-19; IACUC Issue No: ZYD-2020-111) and were performed in accordance with the NIH Guide for the Care and Use of Laboratory Animals. Efforts were made to minimize suffering and the number of mice used in the experiments.

### PCR Genotyping

DNA was extracted from mouse tail (0.5–1 mm). PCR reactions were performed with 400 ng DNA template. Amplification program consisted of an initial denaturation at 94°C for 3 min, 35 cycles of denaturation at 94°C for 30 s, annealing at 60°C for 35 s, and extension at 72°C for 35 s. The products were subjected to 2% agarose gel electrophoresis, and bands were imaged using the Omega Lum G imaging system (Aplegen). The primers are listed in Table 1.

**TABLE 1:** Oligonucleotides used in this study.

	Forward (5′-3′ sequence)	Reverse (5′-3′ sequence)
**PCR genotyping**		
*Per2-1*	TAC​TTC​TGA​GTC​CTG​GTT​GTT​CTT​G	ACC​ACA​TTA​CCT​CAA​AGT​CCC​AC
*Per2-2*	AAA​TGG​AGT​TAT​TCA​GAG​GAG​GAA​C	ACC​ACA​TTA​CCT​CAA​AGT​CCC​AC
**qPCR**		
*Cyp2b10*	^99 bp^ AAA​GTC​CCG​TGG​CAA​CTT​CC	^327 bp^ TTG​GCT​CAA​CGA​CAG​CAA​CT
*Sult1a1*	^137 bp^ CAC​AAG​GGT​CCT​CTC​CTT​AGC	^239 bp^ CCA​GAC​TTT​GGG​TAC​GTG​CT
*Ugt1a9*	^492 bp^ TTT​CGA​TGT​GTG​CGG​CTT​AAC	^652 bp^ GGT​TCC​GAG​TTC​TTT​CCT​TGA​A
*Rev-erbα*	^1096 bp^ TTT​TTC​GCC​GGA​GCA​TCC​AA	^1272 bp^ ATC​TCG​GCA​AGC​ATC​CGT​TG
*Per2-*primer1	^636 bp^ GCT​GCT​AAT​GTC​CAG​TGA​G	^845 bp^ AGCCAGGAACT CCACAAACT
*Per2-*primer2	^2960 bp^ CCA​CAC​TTG​CCT​CCG​AAA​TA	^3095 bp^ ACT​GCC​TCT​GGA​CTG​GAA​GA
*Ppib*	^45 bp^ TCC​ACA​CCC​TTT​TCC​GGT​CC	^156 bp^ CAA​AAG​GAA​GAC​GAC​GGA​GC
**siRNA**		
siRev-erbα	UUC​UCC​GAA​CGU​GUC​ACG​UTT	ACG​UGA​CAC​GUU​CGG​AGA​ATT
siPer2	GGA​UAG​AGG​CCC​AGA​CGU​ATT	UAC​GUC​UGG​GCC​UCU​AUC​CTT
siNC	CGA​UUA​GUC​UAU​ACG​UUC​UCC​UGA​G	CUC​AGG​AGA​ACG​UAU​AGA​CUA​AUC​G

### Pharmacokinetic Study

*Per2* ^*Del4-6*^ and control wild-type mice (8–12 weeks, male) were treated with a single dose of CPA (100 mg/kg) by intraperitoneal injection at ZT14. At predetermined time points (15, 30, 60 and 120 min), mice (*n* = 5 per time point) were rendered unconscious with isoflurane, and plasma samples were collected by retro-orbital bleeding. Plasma samples were processed for UPLC-QTOF/MS analysis as previously described (Sadagopan et al., 2001; Zhao et al., 2019). Of note, the CPA metabolite, 4-hydroxycyclophosphamide (4-OH-CPA) is unstable in the plasma (Sadagopan et al., 2001). To quantify 4-OH-CPA, plasma samples were treated with O-methylhydroxylamine (OMHA) to transform 4-OH-CPA into a stable product OH-CPA O-methyloxime (Sadagopan et al., 2001). Additionally, liver samples were collected at two time points (i.e., 30 and 120 min), and processed to measure the drug/metabolite concentrations in the livers as previously described (Zhang et al., 2018a).

### Acute Toxicity Study

*Per2* ^*Del4-6*^ and control wild-type mice (8–12 weeks, male, *n* = 5 per group) were injected (i.p.) with CPA at 100 mg/kg or with vehicle at ZT14. Four hours after drug administration, mice were sacrificed to collect plasma and liver samples, followed by biochemical analyses.

### Biochemical Analysis

ALT, AST, and GSH were measured with their assay kits according to manufacturer’s instructions (Jiancheng Bioengineering Institute, Nanjing, China).

### CYP Microsomal Metabolism Assay

Livers were collected from *Per2* ^*Del4-6*^ and control wild-type mice. Liver microsomes were prepared by sequential ultracentrifugation, first at 9,000 g for 10 min and then at 100,000 g for 1 h as previously described (Van et al., 1992). CPA was used as a specific substrate to determine the microsomal activity of CYP2B10 (Pass et al., 2005; Zhao et al., 2019). In brief, liver microsomes (4 mg/ml) was incubated with CPA (20 μM), NADPH (1.5 mM), and MgCl_2_ (5 mM) in potassium phosphate (50 mM, pH 7.4) at 37°C for 2 h. The reaction was terminated by adding ice-cold acetonitrile (containing an internal standard). To quantify 4-OH-CPA, the resultant mixture was treated with OMHA (1.5 mg/ml) to transform 4-OH-CPA into a stable product OH-CPA O-methyloxime. The reaction mixture was centrifuged at 13,000 g for 15 min and the supernatant was subjected to UPLC-QTOF/MS analysis.

### Glucuronidation Assay

Glucuronidation assays were performed by incubating liver microsomes with propofol. The incubation mixture contained liver microsomes (4 mg/ml), propofol (100 μM), MgCl_2_ (0.88 mM), saccharolactone (4.4 mM), alamethicin (11.2 μM) and UDPGA (3.5 mM) in 50 mM potassium phosphate (pH 7.4). After 30 min, the metabolic reaction was terminated by adding 200 μl ice-cold water/acetonitrile (50:50, v/v) (containing an internal standard). The resulting mixture was centrifuged at 2,000 g for 10 min, and the supernatant was subjected to UPLC-QTOF/MS analysis.

### Sulfation Assay

Livers were collected from *Per2* ^*Del4-6*^ and control wild-type mice. Liver S9 fraction was prepared by centrifugation at 9,000 g for 10 min (Richardson et al., 2016). Sulfation activity was determined using an incubation method with liver S9 fraction as previously described (Guo et al., 2018). Briefly, liver S9 fraction (4 mg/ml), PAPS (50 μM) and galangin (6 μM) were incubated in potassium phosphate buffer (50 mM, pH 7.4) at 37°C for 3 h. The reactions were terminated by adding 200 μl ice-cold acetonitrile (containing an internal standard), followed by vortex and centrifugation at 13,000 g for 15 min. The supernatant was subjected to UPCL-QTOF/MS analysis.

### UPLC-QTOF/MS Analysis

Drugs and metabolites were quantified using an UPLC-QTOF/MS system (AB SCIEX, CA) and a Phenomenex C18 column (2 × 100 mm, 1.6 μm; Phenomenex, Torrance). The mobile phases were 0.1% formic acid in acetonitrile (mobile phase A) and 0.1% formic acid in water (mobile phase B). For determination of CPA and OH-CPA O-methyloxime, the gradient elution program was as follows: 0–2 min, 90% B; 2–6 min, 90-10% B; and 6–8 min, 10–90% B. The mass spectrometer was operated at the positive ion full scan mode. For determination of propofol glucuronide, the gradient elution program was as follows: 0–2 min, 80% B; 2–4 min, 80-30% B; and 4–5 min, 30-80% B. For determination of galangin sulfate, the gradient elution program was as follows: 0–1 min, 90%B; 1–3.5 min; 90-10% B; 3.5–4.5 min, 10% B; and 4.5–5 min, 10–90% B. The mass spectrometer was operated at the negative ion full scan mode for propofol glucuronide and galangin sulfate. The flow rate was set at 0.3 ml/min. Peak areas were recorded with extract masses as follows: *m/z* 261.03 ± 0.05 Da for CPA, 306.09 ± 0.05 Da for OH-CPA O-methyloxime; *m/z* 353.16 ± 0.05 Da for propofol glucuronide; and *m/z* 348.9 ± 0.05 Da for galangin sulfate.

### Cell Culture and Transfection

Hepa-1c1c7 cells were cultured in DMEM containing 10% fetal bovine serum (FBS). AML-12 cells were cultured in DMEM/F12 supplemented with 0.1% dexamethasone, 1% ITS (i.e., insulin, transferrin and selenium) and 10% FBS. Cells were seeded into 6-well plates. Once growing to a density of 60–70%, cells were co-transfected with *Per2* expression plasmid (2 μg) or siPer2 (50 nM) or control using jetPRIME transfection reagent (Polyplus Transfection, Illkirch, France) according to the manufacturer’s protocol. After 24 or 48 h, cells were collected for mRNA and protein quantification.

### qPCR Assay

Total RNA was extracted from mouse liver and cell samples with TRIzol reagent (Accurate Biotech, Hunan, China) following the manufacturer’s instructions, and used as a template for cDNA synthesis. qPCR reactions were performed with SYBR Green Master Mix (Vazyme, Nanjing, China) using a Biometra Toptical Thermocycler (Analytik Jena, Goettingen, Germany) as previously described (Yamamura et al., 2010). Amplification reactions consisted of an initial denaturation at 95°C for 5 min, followed by 40 cycles of denaturation at 95°C for 15 s, annealing at 60°C for 30 s, and extension at 72°C for 30 s. *Peptidylprolyl isomerase B* (*Ppib*) was used as an internal control. Relative mRNA expression was calculated using the 2^-∆∆Ct^ method and normalized to the control group. Primer sequences are shown in Table 1.

### Western Blotting

Mouse tissues and cell samples were lysed in RIPA buffer supplemented with a protease inhibitor cocktail (HY-K0010, Monmouth Junction, NJ). Protein concentration was detected by a BCA assay kit (Beyotime, Shanghai, China). After denaturing at 95°C for 10 min, protein samples (40 μg) were subjected to SDS-polyacrylamide gel electrophoresis (10% gel) and transferred to polyvinylidene fluoride membranes (Millipore, Bedford, MA). The membranes were sequentially incubated with primary antibody and HRP-conjugated secondary antibody. Protein bands were visualized using the Omega Lum G imaging system (Aplegen), and band density was analyzed by using FlourChem software (Bio-Rad). Glyceraldehyde-3-phosphate dehydrogenase (GAPDH) was used as a loading control. Protein expression was normalized to GAPDH.

### Luciferase Reporter Assay

HEK293T cells were cultured in DMEM supplemented with 10% FBS and seeded into 48-well plates. Cells were transfected with *Cyp2b10* luciferase (firefly) reporter plasmid (50 ng), pRL-TK (15 ng) and overexpression plasmid (*Per2* or *Rev-erbα,* 200 ng) or blank pcDNA3.1 (control) using jetPRIME transfection reagent (PolyplusTransfection, Illkirch, France). After 24 h, cells were lysed in 45 μl passive lysis buffer (Promega, Madison, WI), and cell lysate was collected to determine the luciferase activities using the Dual-Luciferase Reporter Assay system on a GloMax 20/20 luminometer (Promega). Firefly luciferase activities were normalized to renilla luciferase values, and expressed as relative luciferase units.

### Data Analysis

Data are presented as mean ± SD (standard deviation). Sample sizes are provided in figure legends. Student’s t-test was used to analyze a statistical difference between two groups. One-way ANOVA followed by Bonferroni post hoc test was used for multiple group comparisons. Statistical analyses were performed with GraphPad Prism 7 (GraphPad Software Inc., San Diego, CA). Pharmacokinetic analysis was performed using WinNonlin software (Pharsight Corp, St. Louis, MO). The level of significance was set at *p* < 0.05 (*).

## Results

### Characterization of *Per2*-Deficient Mice

**FIGURE 1:**
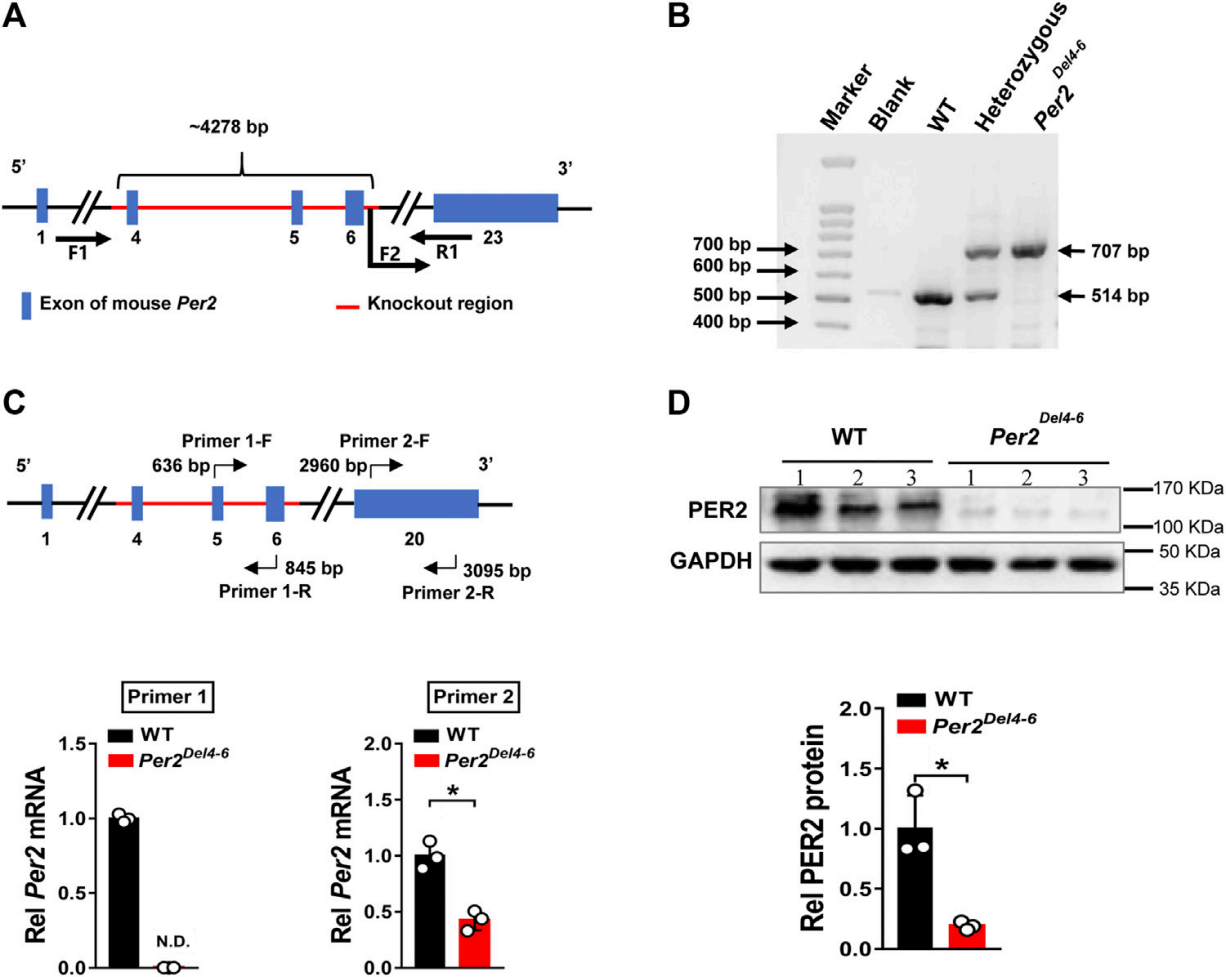
Characterization of Per2-deficient mice. **(A)** Schematic diagram showing target site (exons 4-6) deleted for establishment of *Per2* ^*Del4-6*^ mice. **(B)** PCR genotyping using mouse tails from wild-type (WT), heterozygous and homozygous (*Per2* ^*Del4-6*^) mice. **(C)** qPCR analyses of *Per2* in *Per2* ^*Del4-6*^ and WT mice. The top panel shows the target sites of primer sets 1 and 2. **(D)** Western blotting analyses of PER2 protein in *Per2* ^*Del4-6*^ and WT mice. Western blot strips (a target protein and a loading control) were cut from one gel. Data are mean ± SD (*n* = 3). *p < 0.05 (t test).

*Per2-*deficient mice with exons 4-6 deleted (named *Per2* ^*Del4-6*^ mice) were obtained from Cyagen Biosciences (Guangzhou, China) (Figure 1A). PCR genotyping of mice was performed using tail biopsies with the primers F1, F2 and R1/2, resulting in a 707 bp fragment for *Per2* ^*Del4-6*^ mice and a 514 bp fragment for wild-type allele (Figure 1B). qPCR assay with primer set 1 (targeting the knockout sequence) confirmed the absence of wild-type *Per2* transcript in mouse liver (Figure 1C). Western blotting showed that hepatic PER2 protein was markedly reduced in *Per2* ^*Del4-6*^ mice, however, it was not completely lost (Figure 1D). qPCR assay with primer set 2 targeting non-knockout sequence suggested the presence of a new version of *Per2* transcript in *Per2* ^*Del4-6*^ mice albeit at a reduced level (Figure 1C). This new transcript may be translated to a PER2-related protein that retains a reactivity with the commercial PER2 antibody, thereby explaining why PER2 protein was detected in *Per2* ^*Del4-6*^ mice (Figure 1D). Taken together, *Per2* ^*Del4-6*^ mice were characterized with loss of wild-type *Per2* transcript and markedly reduced PER2 protein.

### Altered Expression of Drug-Metabolizing Enzymes in *Per2* ^*Del4-6*^ Mice

**FIGURE 2:**
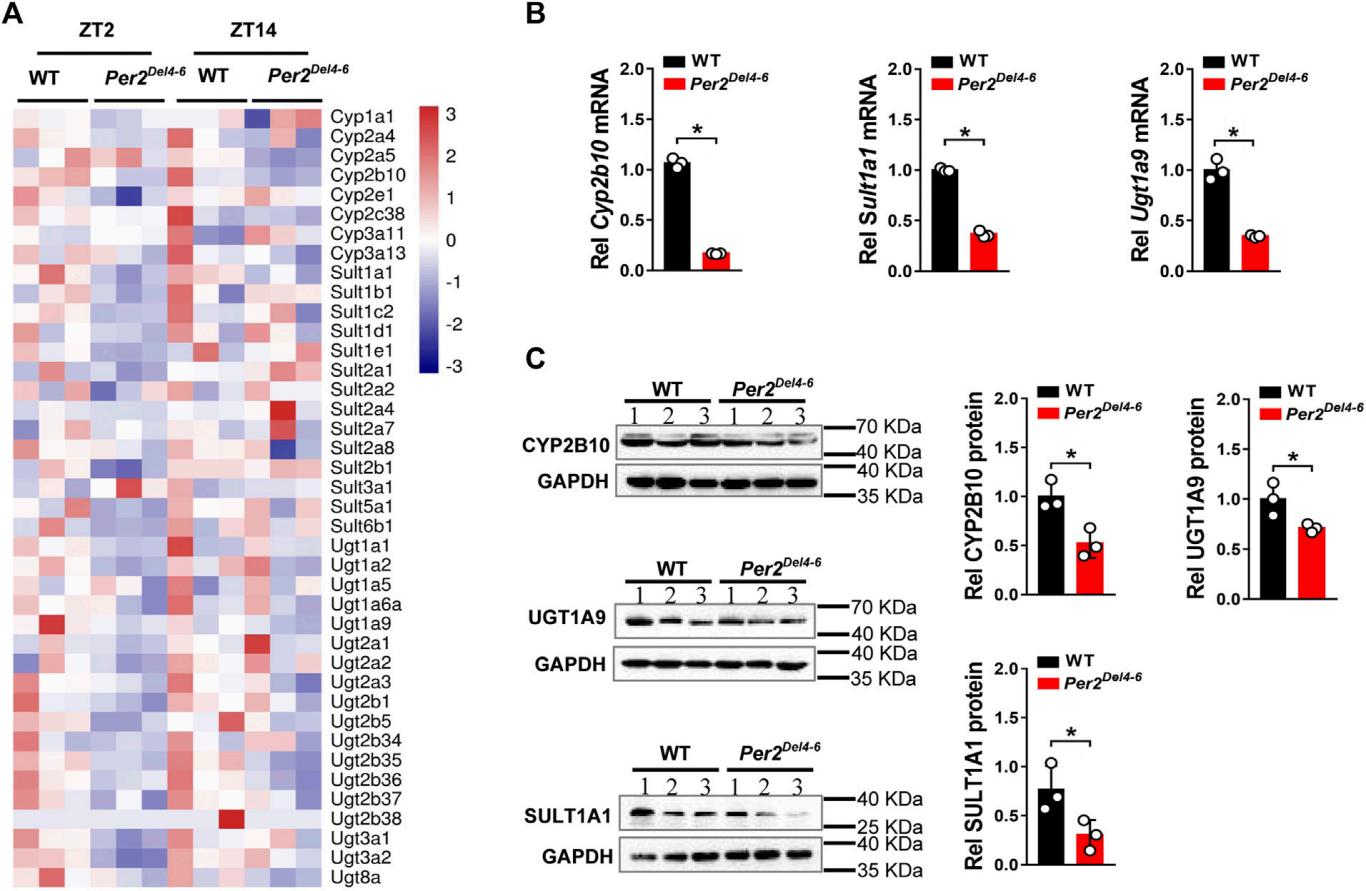
Expression of selected drug-metabolizing enzymes in Per2^Del4-6^ mice. **(A)** Heatmap showing relative mRNA expression of drug-metabolizing enzymes at ZT2 and ZT14 in *Per2* ^*Del4-6*^ versus in WT (wild-type) mice. **(B)** mRNA expression levels of three drug-metabolizing genes (*Cyp2b10, Ugt1a9* and *Sult1a1*) in the livers of *Per2* ^*Del4-6*^ and WT mice. **(C)** Protein expression levels of CYP2B10, UGT1A9 and SULT1A1 in the livers of *Per2* ^*Del4-6*^ and WT mice. Western blot strips (a target protein and a loading control) were cut from one gel. Data are mean ± SD (*n* = 3). *p < 0.05 (t test). Rel, Relative. ZT, zeitgeber time.

**FIGURE 3:**
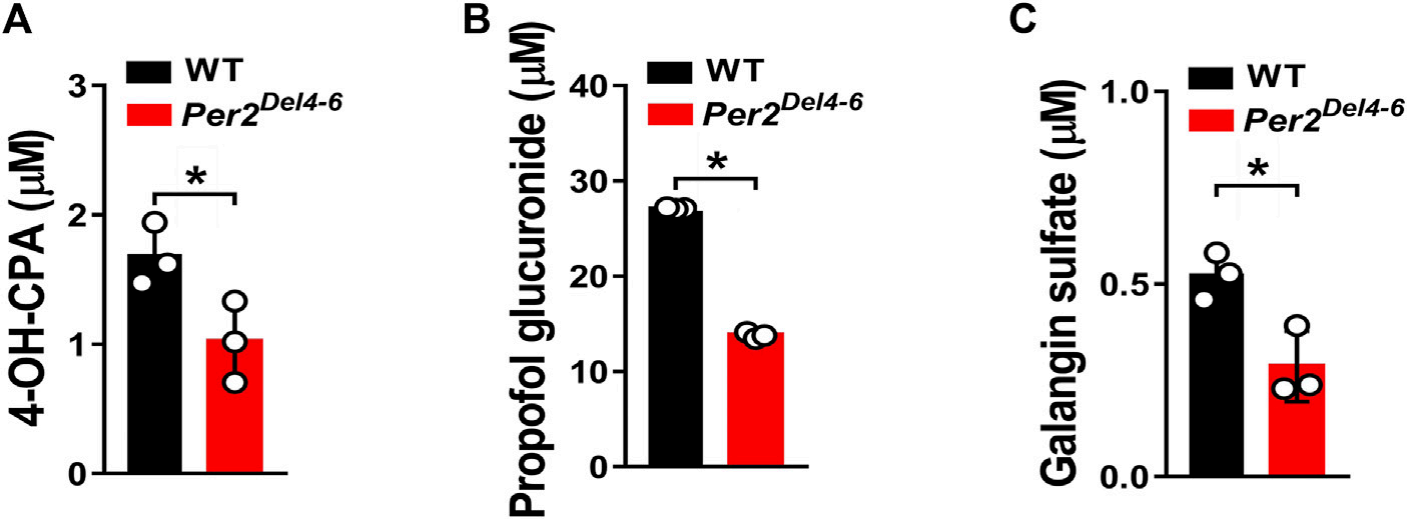
Metabolic activities of CYP2B10, UGT1A9 and SULT1A1 in Per2^Del4-6^ mice based on in vitro metabolism assays. **(A)** Liver CYP microsomal metabolism assays showing attenuated metabolism of CPA in *Per2* ^*Del4-6*^ mice. **(B)** Reduced glucuronidation of propofol in liver microsomes of *Per2* ^*Del4-6*^ mice. **(C)** Sulfation assay showing reduced hepatic metabolism of galangin in *Per2* ^*Del4-6*^ mice. Data are mean ± SD (*n* = 3). *p < 0.05 (t test).

*Per2* expression displays a robust diurnal oscillation with the nadir at ZT2 and the zenith at ZT14 (Yamamura et al., 2010; Yoo et al., 2017). Therefore, the impact of Per2 on expression of drug-metabolizing genes was assessed at two diurnal time points (i.e., ZT2 and ZT14) using *Per2* ^*Del4-6*^ mice. According to qPCR analyses, hepatic expression of several drug-metabolizing genes (including *Cyp2a4/2a5*, *Cyp2b10, Ugt1a1, Ugt1a9, Ugt2b36, Sult1a1,* and *Sult1e1*) was altered (and majority were down-regulated) at both ZT2 and ZT14 in *Per2* ^*Del4-6*^ mice (Figure 2A)*.* Of note, *Cyp2b10, Ugt1a9,* and *Sult1a1* were three genes considerably affected, independent of diurnal times (Figure 2B). Consistent with the mRNA changes, the proteins of CYP2B10, UGT1A9 and SULT1A1 in the liver were significantly reduced in *Per2* ^*Del4-6*^ mice (Figure 2C). In addition, *Per2* ^*Del4-6*^ mice showed decreased enzymatic activities of CYP2B10, UGT1A9 and SULT1A1 against their specific substrates (CPA for CYP2B10, propofol for UGT1A9, and galangin for SULT1A1) based on *in vitro* incubation assays (Figure 3). Taken together, these data revealed a role of *Per2* in regulation of drug-metabolizing enzymes.

### Altered Metabolism and Pharmacokinetics of CPA in *Per2* ^*Del4-6*^ Mice

**FIGURE 4:**
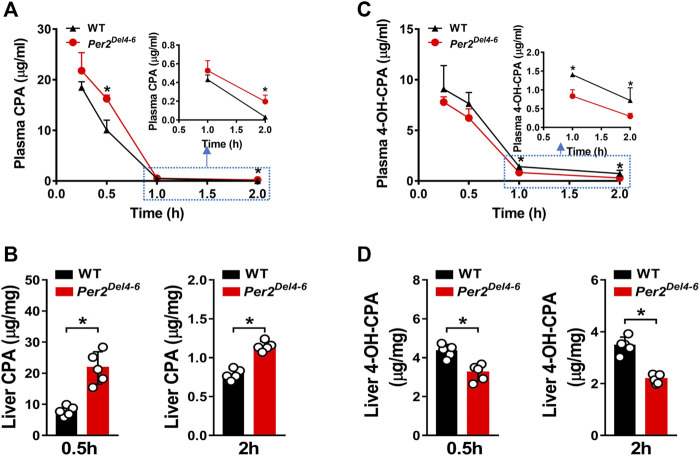
Characterization of CPA metabolism and pharmacokinetics in Per2^Del4-6^ mice. **(A)** Plasma concentrations of CPA in *Per2* ^*Del4-6*^ and WT mice at different time points after CPA treatment (i.p., 100 mg/kg). **(B)** Plasma concentrations of 4-OH-CPA in *Per2* ^*Del4-6*^ and WT mice at different time points after CPA treatment. **(C)** Liver concentrations of CPA in *Per2* ^*Del4-6*^ and WT mice at 0.5 and 2 h after CPA treatment. **(D)** Liver concentrations of CPA in *Per2* ^*Del4-6*^ and WT mice at 0.5 and 2 h after CPA treatment. Data are mean ± SD (*n* = 5). *p < 0.05 (t test).

Since CYP2B10 expression was altered in the liver of *Per2* ^*Del4-6*^ mice, we next tried to test whether this affects the metabolism and pharmacokinetics of substrate drugs. To this end, we performed pharmacokinetic experiments of CPA with *Per2* ^*Del4-6*^ and control mice. *Per2* ^*Del4-6*^ mice showed increases in plasma CPA concentrations (Figure 4A). Accordingly, AUC (area under the curve, a measure of systemic exposure) of CPA was significantly increased in *Per2* ^*Del4-6*^ mice (Table 2). In the meantime, liver CPA concentrations were increased in *Per2* ^*Del4-6*^ mice (Figure 4B). By contrast, the plasma and hepatic levels of the metabolite 4-hydroxycyclophosphamide (4-OH-CPA) were lower in *Per2* ^*Del4-6*^ mice, suggesting reduced metabolism of CPA (Figures 4C,D). Therefore, *Per2* ablation altered the pharmacokinetics of CPA in mice by down-regulating drug metabolism.

**TABLE 2:** Pharmacokinetic parameters for CPA (100 mg/kg, i.p.) after drug administration to *Per2* ^*Del4-6*^ and wild-type (WT) mice.

Parameter	Unit	WT	*Per2* ^*Del4-6*^
C_max_	μg/ml	18.47 ± 0.66	21.77 ± 0.21
AUC	μg/ml^a^h	8.78 ± 0.56	12.02 ± 0.13^a^
MRT	H	0.39 ± 0.03	0.43 ± 0.03
CL/F	(mg/kg)/(μg/ml)/h	11.48 ± 0.76	8.27 ± 0.09^a^

a *p* < 0.05 versus WT.

**FIGURE 5:**
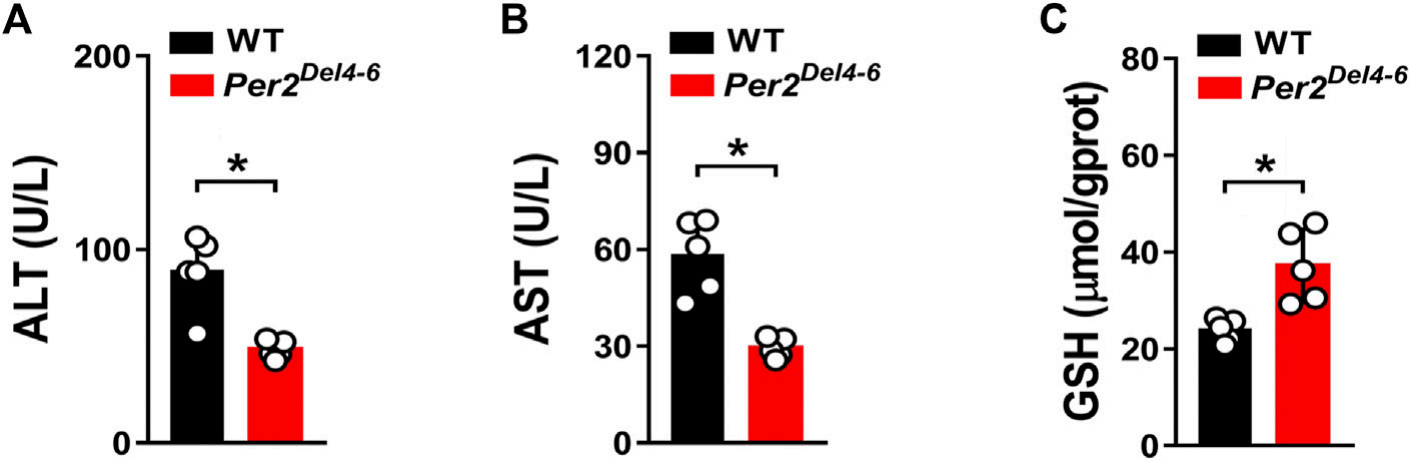
Characterization of CPA-induced hepatotoxicity in Per2^Del4-6^ mice. **(A)** Plasma ALT levels in *Per2* ^*Del4-6*^ and WT mice at 4 h after CPA administration (i.p., 100 mg/kg, *n* = 5). **(B)** Plasma AST levels in *Per2* ^*Del4-6*^ and WT mice at 4 h after CPA administration. **(C)** Hepatic GSH levels in *Per2* ^*Del4-6*^ and WT mice at 4 h after CPA administration. Data are mean ± SD (*n* = 5). *p < 0.05 (t test).

CPA is an anticancer prodrug whose cytotoxic effects depend on metabolic activation to its metabolites such as 4-OH-CPA (Richardson et al., 2016). Our previous study shows that CPA treatment induces hepatotoxicity in mice, which is positively related to formation of 4-OH-CPA (Zhao et al., 2019). We thus examined whether CPA-induced hepatotoxicity is affected in *Per2* ^*Del4-6*^ mice. We found lower levels of ALT and AST activities, but a higher GSH level in the liver of *Per2* ^*Del4-6*^ mice, indicating reduced hepatotoxicity in the genetically modified mice (Figure 5). This was consistent with reduced metabolism of CPA and lowered formation of 4-OH-CPA (Figure 4). Altogether, CPA hepatotoxicity is reduced in *Per2* ^*Del4-6*^ mice due to decreased metabolism.

### *Per2* Positively Regulates CYP2B10 Expression in Hepa-1c1c7 and AML-12 Cells

**FIGURE 6:**
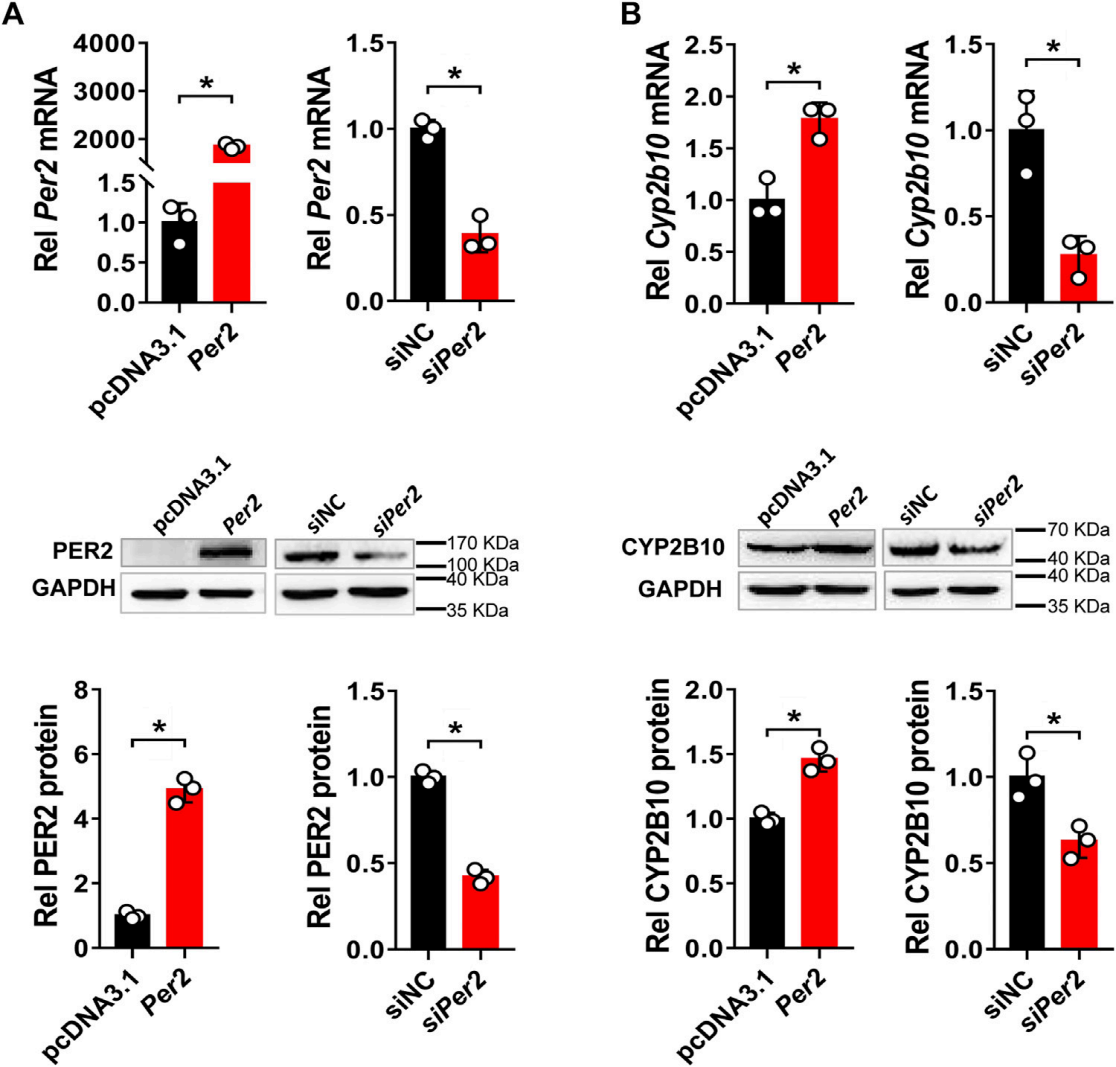
Per2 regulates CYP2B10 expression in Hepa-1c1c7 cells. **(A)** Efficacy validation of *Per2* overexpression (by overexpression plasmid) and knockdown (by siRNA). **(B)** Effects of *Per2* overexpression or knockdown on the expression of CYP2B10 mRNA and protein. Western blot strips (a target protein and a loading control) were cut from one gel. Data are mean ± SD from three independent experiments. *p < 0.05 (t test). Rel, Relative.

**FIGURE 7:**
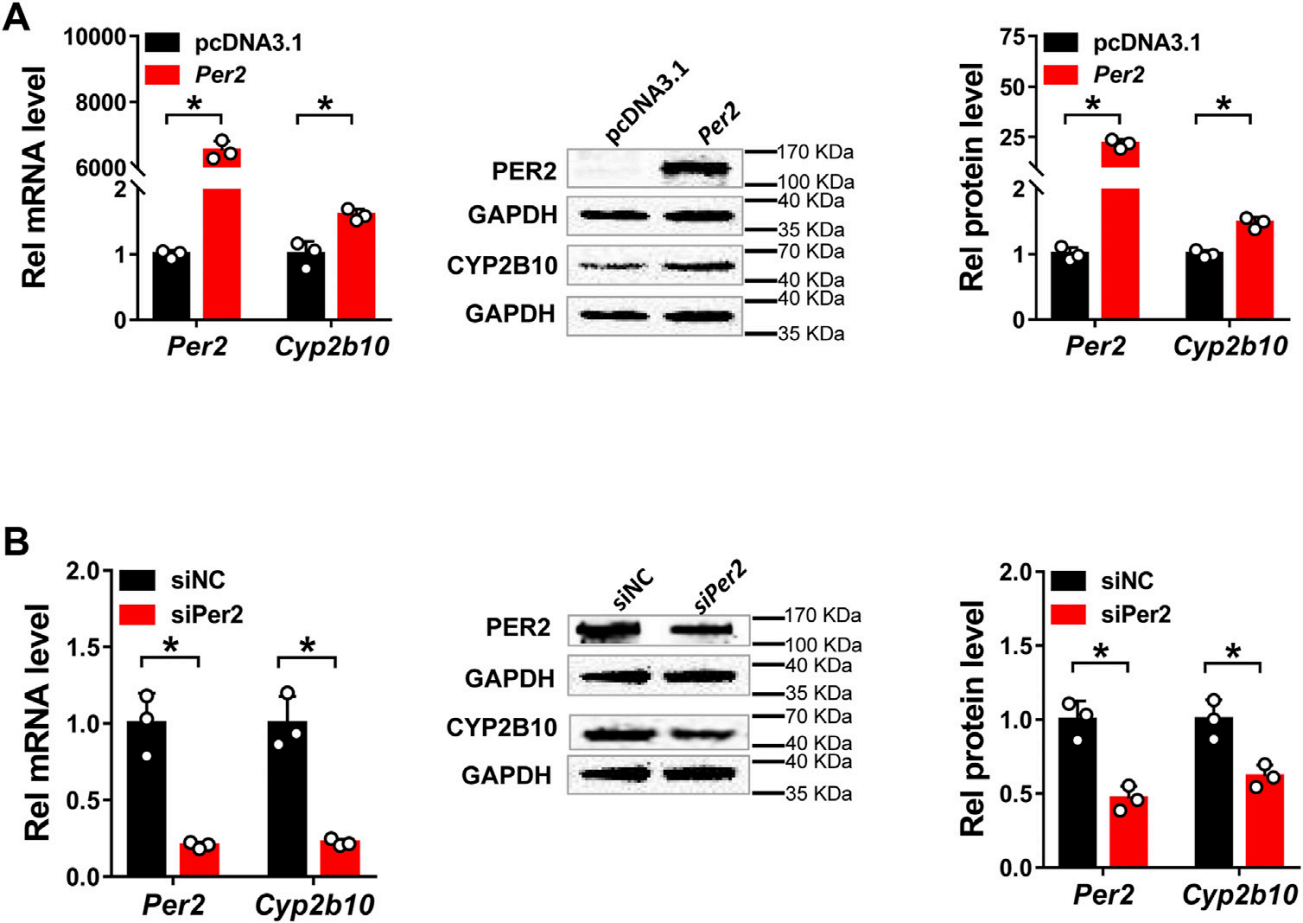
Per2 regulates Cyp2b10 expression in AML-12 cells. **(A)** Effects of *Per2* overexpression on the expression of CYP2B10 mRNA and protein. **(B)** Effects of *Per2* knockdown on the expression of CYP2B10 mRNA and protein. Western blot strips (a target protein and a loading control) were cut from one gel. Data are mean ± SD from three independent experiments. *p < 0.05 (t test). Rel, Relative.

Next, we assessed the regulatory effects of *Per2* on CYP2B10 expression in mouse Hepa-1c1c7 hepatoma cells by performing overexpression and knockdown assays. We confirmed that overexpression plasmid significantly elevated the levels of PER2 mRNA and protein, whereas siRNA decreased PER2 expression, in Hepa-1c1c7 cells (Figure 6A). *Per2* overexpression significantly increased cellular levels of CYP2B10 mRNA and protein (Figure 6B). By contrast, *Per2* knockdown reduced cellular expression of CYP2B10 (Figure 6B). In an identical manner, we examined the effects of *Per2* on CYP2B10 expression in mouse AML-12 hepatocytes (Figure 7). *Per2* overexpression significantly increased CYP2B10 expression, whereas *Per2* knockdown deceased CYP2B10 expression, in AML-12 cells (Figures 7A,B). Taken together, *Per2* positively regulates CYP2B10 expression in both Hepa-1c1c7 and AML-12 cells, consistent with our *in vivo* findings (Figure 2).

### PER2 Regulates CYP2B10 Expression Through REV-ERBα

**FIGURE 8:**
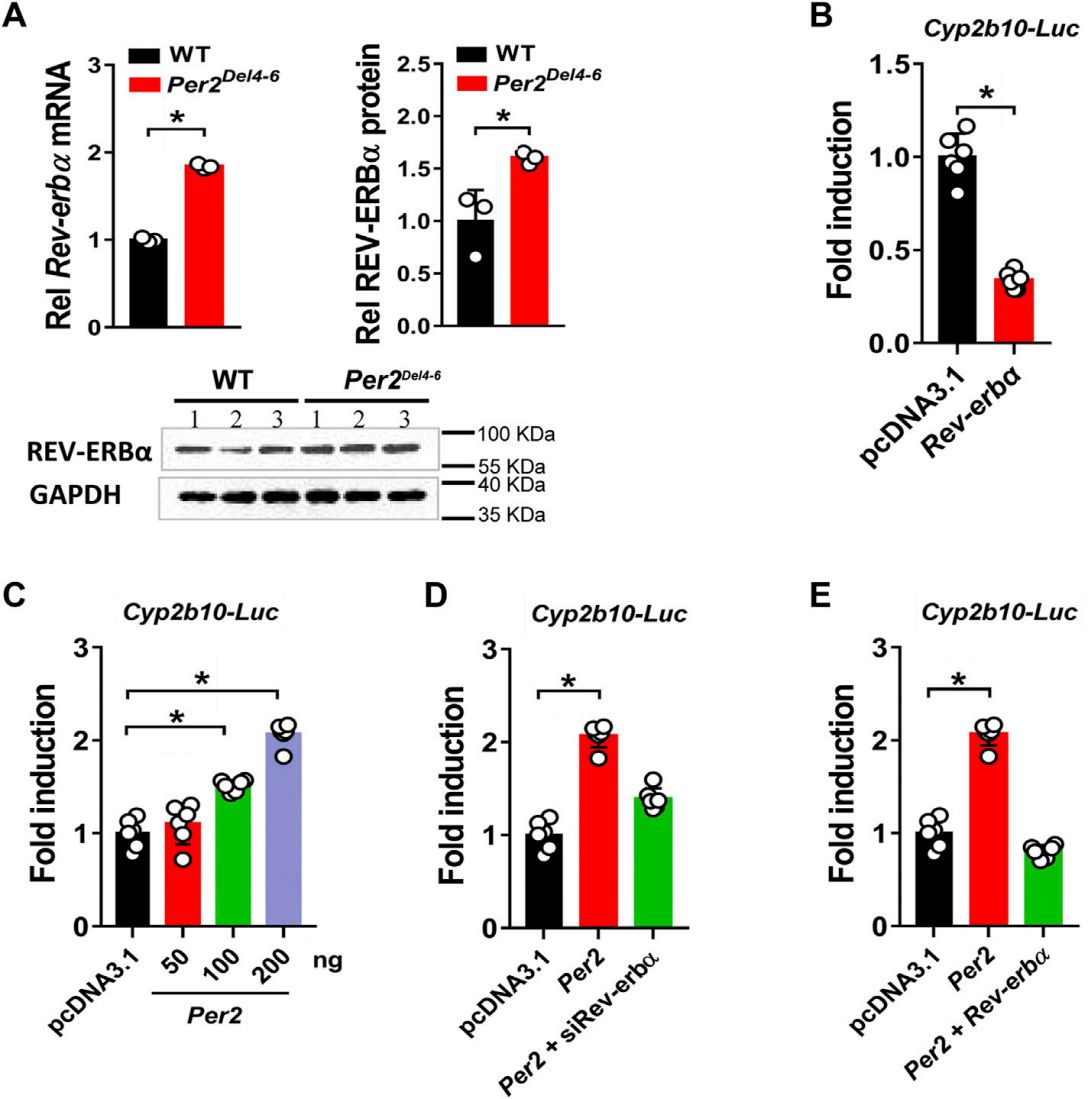
Per2 regulates Cyp2b10 transcription through down-regulating REV-ERBα. **(A)** Increased expression of REV-ERBα in *Per2* ^*Del4-6*^ mice. Western blot strips (a target protein and a loading control) were cut from one gel. **(B)** Effects of *Rev-erbα* overexpression *Cyp2b10*-*Luc* reporter activity in HEK293T cells. **(C)** *Per2* dose-dependently induces *Cyp2b10-Luc* reporter activity in HEK293T cells. **(D)** Regulatory effects of *Per2* overexpression on *Cyp2b10* are attenuated in *Rev-erbα*-deficient HEK293T cells. **(E)** *Rev-erbα* reverses the effects of *Per2* on *Cyp2b10* transcription in HEK293T cells. Data are mean ± SD (*n* = 3 or 6). In panels A/B, *p < 0.05 (t test). In panels **(C–E)**, *p < 0.05 (one-way ANOVA followed by Bonferroni post hoc test). Rel, Relative.

PER2 is an integral component of circadian clock, and functions as a co-repressor to repress the transcriptional activity of the BMAL1/CLOCK heterodimer, a well-known activator of many clock-controlled genes (Langmesser et al., 2008). Thus, it was speculated that PER2 may regulate CYP2B10 expression through an indirect mechanism that involves BMAL1/CLOCK and an intermediate regulator. This intermediate regulator should be a target of BMAL1/CLOCK and a repressor of CYP2B10. A survey of the literature suggested REV-ERBα as a candidate for such intermediate regulator because it transcriptionally inhibits CYP2B10 and the expression itself is directly driven by BMAL1/CLOCK (Dunlap, 1999; Crumbley and Burris, 2011; Zhang et al., 2018b). We therefore tested whether REV-ERBα mediates PER2 regulation of CYP2B10. We found that REV-ERBα mRNA and protein were significantly increased in the liver of *Per2* ^*Del4-6*^ mice (Figure 8A). This was expected because PER2 can repress the expression of REV-ERBα by inhibiting the transcriptional activity of BMAL1/CLOCK (Liu et al., 2008). We confirmed that REV-ERBα is a transcriptional repressor of *Cyp2b10* in HEK293T cells based on luciferase reporter assays (Figure 8B). Intriguingly, PER2 dose-dependently induced the transcription of *Cyp2b10* (Figure 8C). However, the induction effect of PER2 was attenuated in *Rev-erbα* silenced cells (Figure 8D). Moreover, overexpressing *Rev-erbα* reversed the effect of PER2 on *Cyp2b10* transcription (Figure 8E). Taken together, our findings suggest that PER2 regulates CYP2B10 expression by down-regulating REV-ERBα.

## Discussion

In this study, we have revealed that many hepatic DMEs including CYP2B10, UGT1A9 and SULT1A1 are under the control of PER2 in mice (Figures 1–3). We further showed that decreased expression of CYP2B10 was translated to reduced metabolism and altered pharmacokinetics of its substrate drug CPA as well as attenuated CPA hepatotoxicity in *Per2-*deficient (*Per2* ^*Del4-6*^) mice (Figures 4, 5). Positive regulation of CYP2B10 by PER2 was confirmed in both Hepa-1c1c7 and AML-12 cells (Figures 6, 7). Based on luciferase reporter assays, it was shown that PER2 regulated *Cyp2b10* transcription in a REV-ERBα-dependent manner (Figure 8). REV-ERBα is negatively regulated by PER2 (decreased REV-ERBα in *Per2* ^*Del4-6*^ mice, Figure 8) and itself is also a repressor of CYP2B10. Therefore, we propose that PER2 regulates CYP2B10 expression and activity through down-regulating REV-ERBα, thereby impacting the metabolism and pharmacokinetics of substrate drugs.

CYP2B10/CYP2B6 activity is a critical determinant to the pharmacokinetics and efficacy of many drugs including CPA, ifosfamide, ketamine, pethidine, methadone, nevirapine and efavirenz (Ekins et al., 2008). For instance, CYP2B6*6 allele carriers have been linked to increased ifosfamide plasma levels and exacerbated toxicities (Mo et al., 2009). Induction of CYP2B6 by a selective activator (e.g., CITCO) facilitates the bioactivation of CPA to 4-OH-CPA and improves the therapeutic outcome of CHOP (cyclophosphamide–doxorubicin–vincristine–prednisone) chemotherapy against non-Hodgkin lymphoma (Shu et al., 2017). Therefore, identification of PER2 as a novel pharmacokinetic determinant of CYP2B10 substrates may facilitate an increased understanding of varied pharmacokinetics and possibly varied drug toxicity and efficacy in a circadian fashion.

**FIGURE 9:**
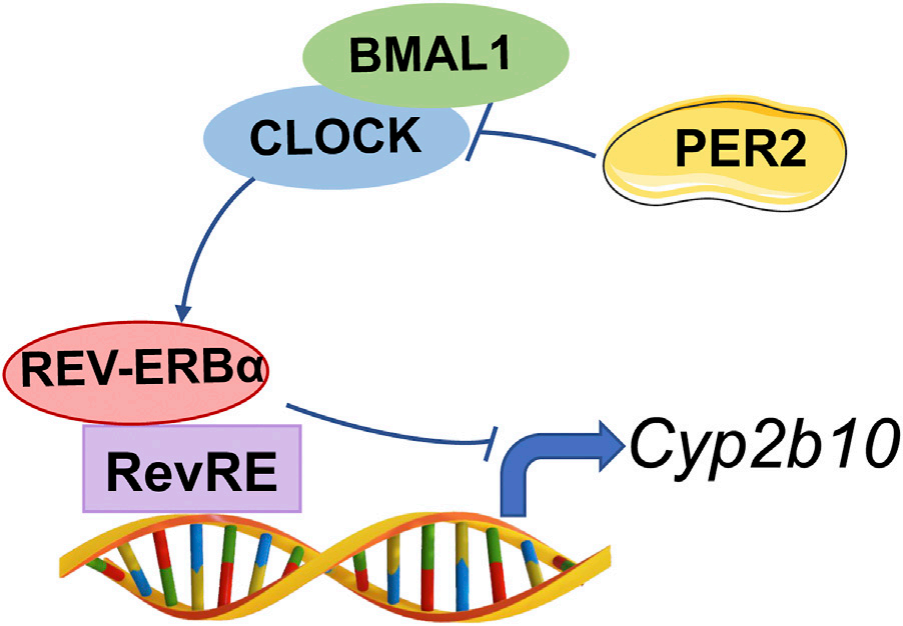
Schematic diagram illustrating the molecular mechanism for PER2 regulation of CYP2B10.

A previous study has revealed *Cyp2b10* as a circadian gene that is under the control of the *Clock* gene (Zhao et al., 2019). It is noteworthy that *Per2* is a circadian clock gene (whose expression oscillates with time of the day) and involved in regulation of circadian rhythms (Yamamura et al., 2010). Therefore, there is a high possibility that *Per2* may be involved in regulation of circadian expression of *Cyp2b10*. However, of clock genes, *Rev-erbα* seems to be a direct transcriptional regulator of *Cyp2b10* and thus a direct key regulator of circadian *Cyp2b10* (Zhang et al., 2018a). Circadian regulation of *Cyp2b10* by other clock genes such as *Per2* and *Clock* is possible, but is attained through modulating *Rev-erbα* (Figure 9). In addition, CAR is another direct transcriptional regulator of *Cyp2b10* (Kettner et al., 2016)*.* However, CAR should not be involved in PER2 regulation of *Cyp2b10* as it positively regulates *Cyp2b10* expression (Kettner et al., 2016). Our findings may provide increased understanding of the complex regulatory pathways for CYP2B10, and highlight the role of clock genes in regulating drug metabolism and pharmacokinetics.

It is noteworthy that we detected a new version of *Per2* transcript in *Per2* ^*Del4-6*^ mice albeit at a reduced level (Figure 1C). This new *Per2* transcript most likely is a truncated version lacking exons 4-6 transcribed region, and may be translated to a PER2-related protein that retains a reactivity with the commercial PER2 antibody, thereby explaining why PER2 protein was detected in *Per2* ^*Del4-6*^ mice (Figure 1D). It is acknowledged that whether or not this PER2-related protein is functional remains unknown. However, it was of no concern that *Per2* ^*Del4-6*^ mice can be used to determine the regulatory effects of *Per2* on hepatic DME *in vivo* as they were characterized with loss of wild-type *Per2* transcript and markedly reduced PER2 protein in the liver (Figure 1).

CPA is a prodrug and bioactivated by CYP2B10 in mice to the hydroxylated metabolite 4-OH-CPA, one active form with cytotoxic effects and hepatotoxicity (Sadagopan et al., 2001). 4-OH-CPA is unstable in biological samples (Sadagopan et al., 2001). To quantify 4-OH-CPA, the samples were treated with OMHA to transform 4-OH-CPA into an equivalent stable product OH-CPA O-methyloxime for mass spectrometric quantification as previously noted (Sadagopan et al., 2001). Alleviated hepatotoxicity of CPA in *Per2* ^*Del4-6*^ mice supported reduced metabolism of CPA to 4-OH-CPA caused by decreased expression of CYP2B10 (Figures 4, 5). Also, reduced CPA metabolism was consistent with decreased systemic clearance (CL/F) according to the pharmacokinetic analysis (Table 2).

Current study focused on determination of the regulatory effects of PER2 on hepatic CYP2B10 and drug pharmacokinetics. It is noteworthy that CYP2B10 expression in other drug-metabolizing organs such as the kidney and intestine besides the liver may be also under the control of PER2 in mice, however, a potential role of renal and intestinal CYP2B10 in altered CPA pharmacokinetics remains unresolved. In addition, we have provided *in vitro* and *in vivo* evidence that hepatic PER2 regulated CYP2B10 in mice to alter pharmacokinetics and drug toxicity. However, whether PER2 regulates CYP2B6-mediated pharmacokinetics and drug toxicity in humans as its counterpart does in mouse liver awaits further investigations.

In summary, CYP2B10 was down-regulated at the mRNA, protein, and enzymatic levels in *Per2*-deficient mice. Consistently, PER2 positively regulated CYP2B10 expression in both Hepa-1c1c7 and AML-12 cells. Moreover, PER2 regulated *Cyp2b10* transcription in a REV-ERBα-dependent manner. Therefore, PER2 regulates CYP2B10 expression and activity through REV-ERBα, impacting the metabolism and pharmacokinetics of substrate drugs.

## Data Availability

The original contributions presented in the study are included in the article/Supplementary Material, further inquiries can be directed to the corresponding authors.
