# Supplementary material for: Period 2 Regulates CYP2B10 Expression and Activity in Mouse Liver
Source: Front Pharmacol. 2021 Nov 23;12:764124. doi: 10.3389/fphar.2021.764124 (PMC8650840; doi:10.3389/fphar.2021.764124)
Supplement: Supplementary file 1 [file DataSheet1.PDF]

We help you discover life

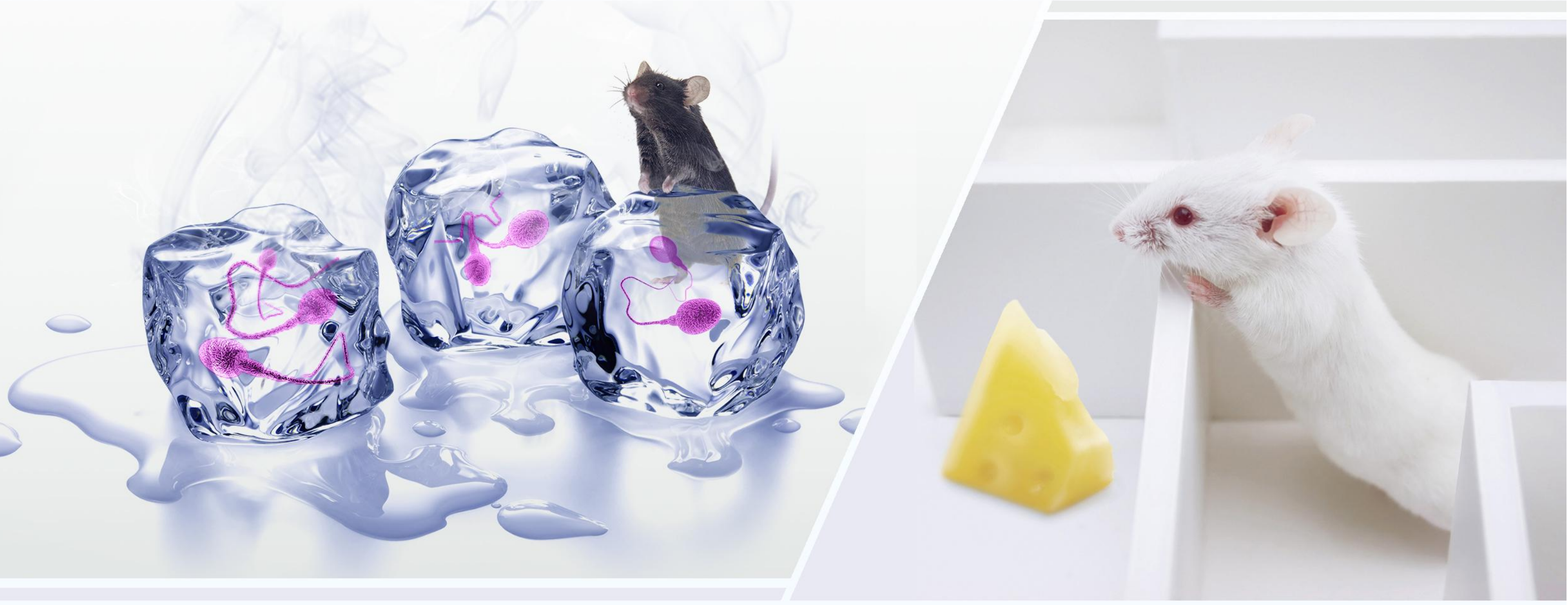

CRISPR-AI Mouse Sperm Bank

Mouse Conventional Knockout User Manual

Contract No.: KOAI181030DA3

- Confidential -

Cyagen Biosciences Inc.
www.cyagen.com

1. Product Information

Name	C57BL/6-Per2^{tm1cyagen}
Serial Number	KOCMP-21210-Per2
Gene	Per2
NCBI ID	18627
Strain	C57BL/6
Type	conventional knockout
Linker	https://www.cyagen.com/cn/zh-cn/sperm-bank/18627

2. gRNA target sequence

gRNA1 (matching forward strand of gene): AAGCCTACACCACTGTACCGGGG
gRNA2 (matching forward strand of gene): TCACTAGCAAGTGTATACGCTGG

3. Delivery Information

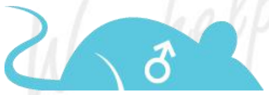

QTY :2
DOB :02-19-2019
ID :1,2

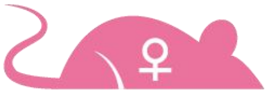

QTY :1
DOB :02-19-2019
ID :6

4. Genotyping Strategy

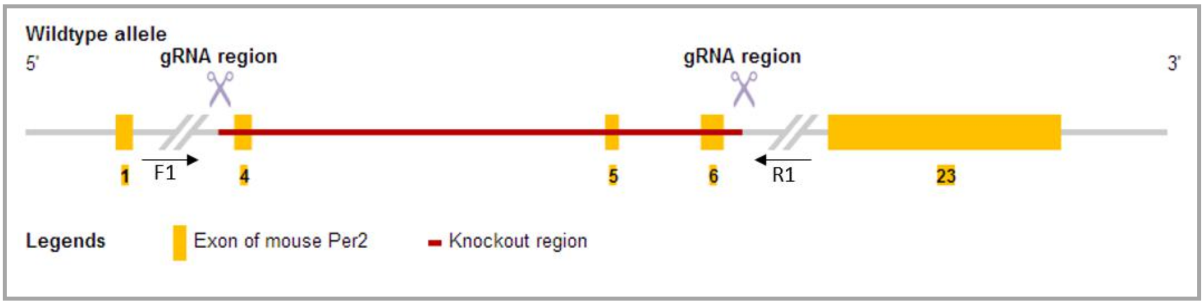

5. PCR Screening

PCR Primers (Annealing Temperature 60.0 ºC):

Forward primer (F1): 5'-TACTTCTGAGTCCTGGTTGTTCTTG-3'
Reverse primer (R1): 5'-ACCACATTACCTCAAAGTCCCAC-3'

Targeted allele: 707 bp Wildtype allele: 5779 bp

PCR Results:

Animals 1, 2 and 6 were identified positive by PCR screening.

Marker **PCR analysis (MT: 707 bp; WT: 5779 bp)**

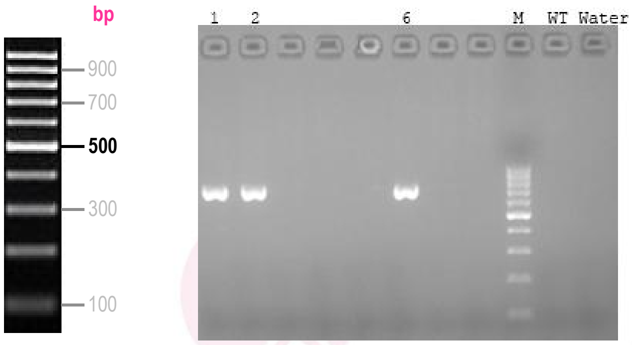

Note:

1) PCR was carried out in 25 μL volume for 35 cycles under standard conditions, with primers listed above added to each reaction.
2) Taq DNA polymerase used was P112-01.
3) Two controls used in PCR genotyping are:

- Water control: No DNA template added.
- Wildtype control: 400 ng of mouse genomic DNA.

6. Sequencing Confirmation

Sequencing Primer for PCR product:

Sequence primer (R1): 5'-ACCACATTACCTCAAAGTCCCAC-3'

Sequencing Results:

Positive animals

Mouse ID: 1, 2, 6 (Deleted 5072 bp)

GGGAAGGAAGAACCCTGTGGTGAAGCCTACACCACTGTAC--del 5072 bp--GCTGGCTTTGTGACCCACCTGTGCCCAGGCTTCTTGCCCT

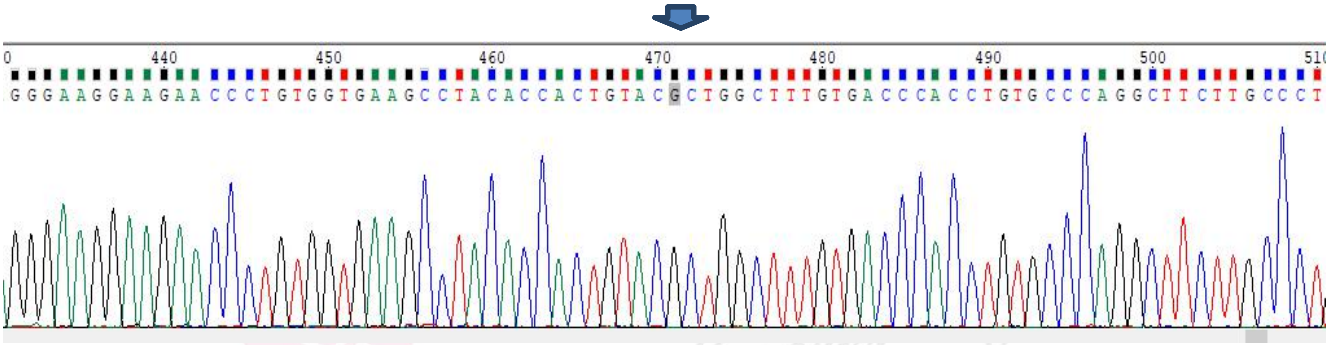

7. Breeding and Genotyping strategy

7.1 Targeting Strategy

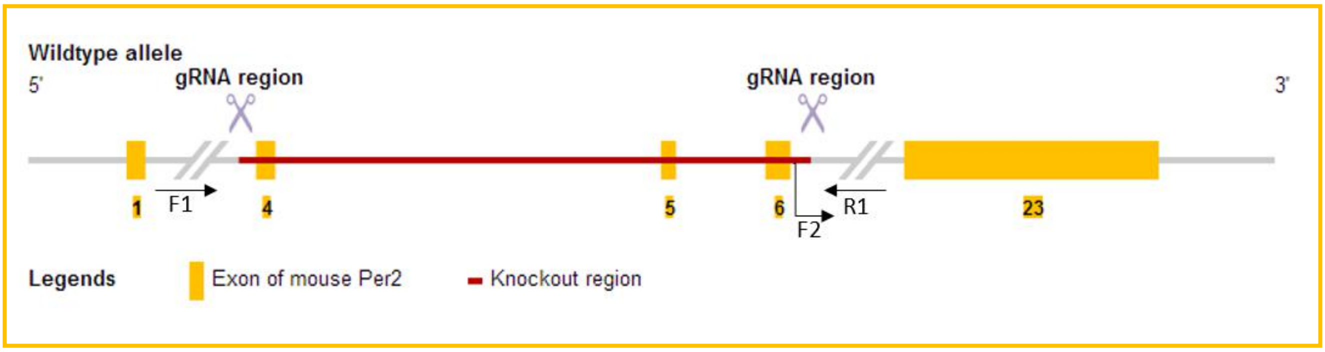

7.2 Cyagen Delivered

Heterozygous recombinant mice

7.3 Method

Inter-cross heterozygous targeted mice to generate homozygous targeted mice

Primers:
F1: 5’-TACTTCTGAGTCCTGGTTGTTCTTG-3’
R1: 5’-ACCACATTACCTCAAAGTCCCAC-3’
F2: 5’-AAATGGAGTTATTCAGAGGAGGAAC-3’

Homozygotes: 707 bp

Heterozygotes: 707 bp/514 bp

Wildtype allele: 514 bp

8. PCR Conditions Attachment

8.1 DNA Extraction

- Method One:

We recommend that using TaKaRa MiniBEST Universal Genomic DNA Extraction kit (Ver.5.0_Code No. 9765) to gain high purity of genomic DNA.

a. Add 180 μL of Buffer GL, 20 μL of Proteinase K and 10 μL of RNase A per tail piece (2-5 mm) in a microcentrifuge tube. Be careful not to cut too much tail.

b. Incubate the tube at 56℃ overnight.

c. Spin in microcentrifuge at 12,000 rpm for 2 minutes to remove impurities.

d. Add 200 μL Buffer GB and 200 μL absolute ethyl alcohol with sufficient mixing.

e. Place the spin Column in a collection tube. Apply the sample to the spin and centrifuge at 12,000 rpm for 2 min. Discard flow-through.

f. Add 500 μL Buffer WA to the spin column and centrifuge at 12,000 rpm for 1 min. Discard flow-through.

g. Add 700 μL Buffer WB to the spin column and centrifuge at 12,000 rpm for 1 min. Discard flow-through. (Note: Make sure the Buffer WB has been premixed with 100% ethanol. When adding Buffer WB, add to the tube wall to wash off the residual salt.)

h. Repeat step g.

i. Place the spin Column in a collection tube and centrifuge at 12,000 rpm for 2 min.

j. Place the spin Column in a new 1.5ml tube. Add 50~200 μL sterilized water or elution buffer to the center of the column membrane and let the column stand 5min. (Note: Heating sterilized water or elution buffer up to 65℃ can increase the yield of elution.)

k. To elute DNA, centrifuge the column at 12,000 rpm for 2 min. To increase the yield of DNA, add the flow-through and/or 50~200 μL sterilized water or elution buffer to the center of the spin column membrane and let the column stand 5 min. Centrifuge at 12,000 rpm for 2 min.

l. Quantify to genomic DNA. Eluted genomic DNA can be quantified by electrophoresis or electrophoresis.

- Method Two:

A low-cost and sample method to gain rough genomic DNA.

Cyagen Biosciences
Tel: 400-680-8038
Email: info@cyagen.com

a. Add 100 µL of tail digestion buffer per tail piece (2-5 mm) in a microcentrifuge tube. Be careful not to cut too much tail.

b. Incubate the tube at 56℃ overnight.

c. Incubate the tube at 98℃ for 13 minutes to denature the Proteinase K.

d. Spin in microcentrifuge at top speed for 15 minutes. Use an aliquot of supernatant straight from the tube (2 µL in a 50 µL reaction) for PCR.

Final concentration of tail digestion buffer:

- 50 mM KCl
- 10 mM Tris-HCl (pH 9.0)
- 0.1 % Triton X-100
- 0.4 mg/mL Proteinase K

8.2 PCR Mixture (primer concentration: 10µM):

Component	x1
ddH_2O	9.0 µl
Product primer F	1.0 µl
Product primer R	1.0 µl
Premix Taq	12.5 µl
DNA	1.5 µl
Total	25 µl

8.3 PCR Reaction Conditions:

Step	Temp.	Time	Cycles
Initial denaturation	94 °C	3 min	
Denaturation	94 °C	30 s	35 x
Annealing	60 °C	35 s	
Extension	72 °C	35 s	
Additional extension	72 °C	5 min	

8.4 Relevant Reagents:

**Trizma Hydrochloride Solution**	Sigma, Cat. No. T2663
**Proteinase K**	Merck, Cat. No. MK539480
**Triton X-100**	Sigma, T8787-50 mL
**2 × Taq Master Mix (Dye Plus)**	Vazyme, P112-01
**Agarose**	BIOWEST AGAROSE, REGULAR
**DNA Marker**	Thermo Scientific GeneRuler 100 bp DNA Ladder #SM0242
**0.5×TBE**	Tris Bio Basic Inc, TBO194-500g
	EDTA Shanghai Sangon, 0105-500g
	Boric Acid, Shanghai Sangon, 0588-500g
